# A qualitative analysis of a psychoeducational video resource to reduce stigma for those who hear voices: The compassion focused therapy ‘Stuart video’

**DOI:** 10.1111/papt.70099

**Published:** 2026-08-18

**Authors:** James N. Kirby, Kaedee Abel, Charles Heriot‐Maitland

**Affiliations:** ^1^ Compassionate Mind Research Group, School of Psychology The University of Queensland Brisbane Australia; ^2^ Department of Psychology King's College London UK; ^3^ Balanced Minds London UK

**Keywords:** compassion focused therapy, psychoeducation, psychosis, stigma, voice hearing

## Abstract

**Objectives:**

Societal stigma towards individuals who hear voices (auditory verbal hallucinations) is a significant barrier to help‐seeking and has been resistant to traditional anti‐stigma campaigns. In this context, Compassion Focused Therapy (CFT) presents a promising alternative approach.

**Design:**

This qualitative study explores the views of people with lived experience of hearing voices regarding a 5‐min psychoeducational video, ‘Compassion for Voices: A Tale of Courage and Hope’, which was designed as a therapeutic and destigmatising resource.

**Method:**

An online survey method was used to recruit participants who identified as having a lived experience of hearing voices. Thematic analysis was conducted on open‐ended survey responses from 20 participants who identified as having a lived experience of hearing voices. The survey followed participants' viewing of the ‘Compassion for Voices’ video, which was co‐produced with individuals who have a lived experience of voice hearing.

**Results:**

The analysis revealed three key themes: ‘A Sense of Shared Identity to Stuart's Journey’, ‘CFT approach desired, but recognition of fears, blocks and resistances to developing compassion’ and ‘Stuart's Psychoeducation Video as Validating, Destigmatising and Hopeful’. Participants largely found the video to be an accurate and validating representation of their experiences. The video was also perceived as a possible resource that could be used to address public stigma.

**Conclusion:**

While preliminary, these findings suggest the video is a useful psychoeducational tool, has the potential to reduce stigma and is viewed as an acceptable intervention. Further research is needed to explore its long‐term impact and use in clinical settings.

**Practitioner Points**

- The ‘Compassion for Voices’ video may serve as a useful resource for fostering validation and reducing feelings of isolation among voice hearers by framing voice hearing as a shared, highly variable human experience.
- Clinicians could use the video during therapy as an accessible, visual introduction to core CFT concepts and the three circle model.
- The video conveys themes of hope and agency, which are recognised as important components of mental health. Clinicians can leverage this material to help clients explore possibilities for living a meaningful life alongside their voice‐hearing experiences.

Hearing voices (also termed auditory verbal hallucinations) without the presence of an external stimulus is often associated with an underlying psychopathology, particularly disorders classified as schizophrenia spectrum or psychosis‐related disorders (Parker, Schnackemberh, & Hopfenbeck, 2021). However, hearing voices is not an uncommon experience within the general population. Studies on experiences of hearing voices date back to the 1800's, with a meta‐analysis concluding the median rate of voice hearing across 17 studies to be 13.2% (Beaven et al., 2011), though some estimates have been as high as 84% (Millham & Easton, 1998). Voice hearing is not limited to adult populations and has also been found to occur across the lifespan. For example, among seven‐ to eight‐year‐old children, 9% were identified to have an experience of hearing voices over a one‐year period (Bartels‐Velthuis et al., 2010); among adolescents aged 16 to 19, the prevalence was 10.6% (Kompus et al., 2015). Hearing voices has also been observed as a cross cultural phenomena, documented to have occurred in Maasai women (Myers et al., 2022), in a sample of the Chinese population (Wang Fung et al., 2020), as well as Indian and Ghanaian populations (Luhrmann et al., 2015). When examining the content of voices, individuals tend to identify both positive and negative themes (e.g. Krakvik et al., 2015), and this varies across cultures (Luhrmann et al., 2015).

Hearing voices, while not an uncommon experience in the general population, may form part of an underlying psychopathology. For example, the presence of voices is an example of a positive symptom within psychotic illnesses such as schizophrenia spectrum disorders (also commonly referred to as psychoses) and can also occur within mood disorders such as bipolar affective disorder type 1 and psychotic depression (American Psychiatric Association, 2022). A median estimate of the lifetime prevalence of psychotic illnesses was roughly eight people per thousand (Moreno‐Kustner, Martin, & Pastor, 2018), and psychotic illnesses are associated with significant functional impairment, decreased quality of life (Heinze et al., 2018) and significant financial burden (Chong et al., 2016). Hearing voices is not only found within psychotic illnesses, but also associated with posttraumatic stress disorder (PTSD; Brewin & Patel, 2010; Clifford et al., 2018) and sometimes conceptualised as a dissociative experience (Longden et al., 2012). Hearing voices is also found in individuals who have experienced complex developmental trauma (e.g. Loberg et al., 2019; Peach et al., 2020) and those with personality disorders, particularly borderline personality disorder (BPD; Kingdon et al., 2010).

Research tends to support the notion that hearing voices may be both a normative experience as well as an indication of underlying psychopathology, importantly though critical differences exist in the relation to voices between clinical and non‐clinical populations. Within a clinical population, voice hearing is associated with significant distress, with voices often perceived as dominating and intrusive, and can also be seen as omnipotent and malevolent; for non‐clinical populations, voices are typically viewed as benevolent and helpful (Sorrell et al., 2010), although some also experience voices as self‐critical (Gilbert et al., 2004). It has been identified in the literature that individuals seeking treatment for hearing voices tend to be experiencing high levels of distress related to their experiences (Greenwood et al., 2010). Thus, it is possible the development of a less negative relationship with voices may serve to reduce distress and is also associated with improvements in quality of life (Lincoln et al., 2016; Longden et al., 2018).

Individuals often face barriers to seeking help for concerns of hearing voices. Stigma has been identified as a main barrier among those who accessing psychosis services to seeking treatment (Burke et al., 2016) and appears to occur cross culturally (e.g., Wood et al., 2014). Individuals living with mental illness often face stigmatising misconceptions such as being seen as weak or lazy (e.g., Abdullah &Brown 2020; Link, Yang, Phelan, & Collins, 2004), and there is even further stigma associated with psychotic disorders. For instance, individuals who experience psychotic illnesses are often stereotyped as dangerous, violent and unpredictable and mislabelled as ‘crazy’ (Bowen et al., 2019; Wood et al., 2014); this view is often further reinforced via portrayals in media (Bowen et al., 2019; Yang & Parrott, 2018). Indeed, there is evidence to suggest beliefs that individuals living with a psychotic illness are dangerous or violent has increased from 1996 to 2018 (Pescosolido et al., 2019). Around 90% of individuals with a psychotic illness report having been discriminated against (Thornicroft et al., 2009) and roughly 65% of individuals with psychotic disorders anticipate experiencing stigma (Gerlinger et al., 2013). The stigma associated with psychosis is also thought to create systemic barriers, including difficulties accessing employment, housing or health care (Thornicroft et al., 2016).

There are also further implications for those with psychotic disorders experiencing stigma from society. When stigma is perceived from society, a process known as self‐stigma can occur, in which the individual takes on these negative beliefs as representative of their internalised image (Corrigan et al., 2006; Corrigan & Watson, 2002). In a meta‐analysis, self‐stigma is linked to both delays in seeking treatment, greater depressive symptoms, lower wellbeing, more hospitalisations, lower medication adherence and overall poorer treatment outcomes in addition to a lowered sense of self‐efficacy and lower self‐esteem (Eliasson, McNamee, Swanson, Lawrie, & Schwannauer, 2021). This negative view of self is also suggested to not only occur through experience of stigma, but also due to the content contained within the voices themselves (Wood, Byrne, & Morrison, 2017).

While there have been many campaigns focussed on reducing stigma in mental health diagnoses with some improvements found (e.g., Morgan et al., 2021) when examining the stigma specifically relating to individuals with psychotic disorders, there was little change as a result of such campaigns (Pescosolido et al., 2019). Indeed, Longden and Read (2017) noted that traditional means of stigma reduction, such as biomedical explanations of psychotic disorders, may actually worsen stigma; biomedical explanations have found to increase perceptions such as recovery from a psychotic illness is unlikely (Haslam, 2011) and was related to a need for social distance and increased perceptions of dangerousness (Kvaale et al., 2013).

While dominant psychiatric frameworks have historically contributed to institutional and self‐stigma among individuals who hear voices, several non‐pathologising and community‐led approaches have emerged to address this issue. Notably, the Hearing Voices Network peer support groups provide a safe, non‐judgmental space where shared lived experiences help normalise voice hearing, reduce self‐stigma and foster psychological acceptance (Corstens et al., 2014; Longden et al., 2018; Oakland & Berry, 2015). Building on this foundation the use of compassion and Compassion Focused Therapy (CFT) has also been explored as an additional pathway of reducing stigma and has shown some utility in reducing stigma in differing populations. For example, CFT has been found to be effective in reducing stigma for those living with human immunodeficiency virus (HIV; Skinta et al., 2015) and among a transdiagnostic sample of individuals living with mental illness (McManus et al., 2018). Thus, CFT may warrant exploration for reducing stigma towards those who hear voices.

## COMPASSION FOCUSED THERAPY APPROACH TO VOICE HEARING

CFT is an integrative and evolutionary informed biopsychosocial model of therapy (Gilbert & Simos, 2022). Theoretically CFT is underpinned by social mentality theory, and a key focus of the therapeutic approach is to shift individuals out of competitive social rank focused motivations to that of compassionate motivations (Kirby & Petrocchi, 2023). Meta‐analytic research has found that CFT is effective at reducing negative symptoms and improving positive wellbeing for clinical and non‐clinical populations (Petrocchi, Ottaviani, et al., 2024). A core feature of CFT is its tripartite model of affect regulation commonly referred to as the three circle model (Moloney‐Gibb et al., 2025, 2026) and is depicted in Figure 1. The threat system (red circle) is focused on harm avoidance and protection, as a result is sensitive to social and non‐social dangers and threats. Emotions experienced in the threat system typically include fear, anger and disgust, and it is associated physiologically with sympathetic activity. The drive system (blue circle) is focused on the pursuit of goals (e.g., status) and acquisition of resources (e.g., food, shelter) and activates positive emotions when these are acquired, such as excitement, happiness and joy, which are also associated physiologically with the sympathetic system. The soothing system (green circle) is focused on a different type of positive emotion, those linked with experiences of safeness, connectedness and contentment, and is associated physiologically with the parasympathetic system. These emotion systems interact and operate in accordance with context; thus, in some settings, a dominant threat system with a weaker soothing system is important (e.g., responding appropriately to a crises). However, difficulties with emotion regulation can occur, particularly when dominant threat systems remain even in contexts of safety (e.g., ruminating at home in bed). CFT aims to increase an individual's inner sense of safety, warmth and soothing through use of compassionate mind training exercises and strategies aimed to increase the balance between these systems, typically by increasing the soothing system (green circle) to reduce the activation of the threat system (red circle) (Petrocchi, Ottaviani, et al., 2024).

**FIGURE 1:**
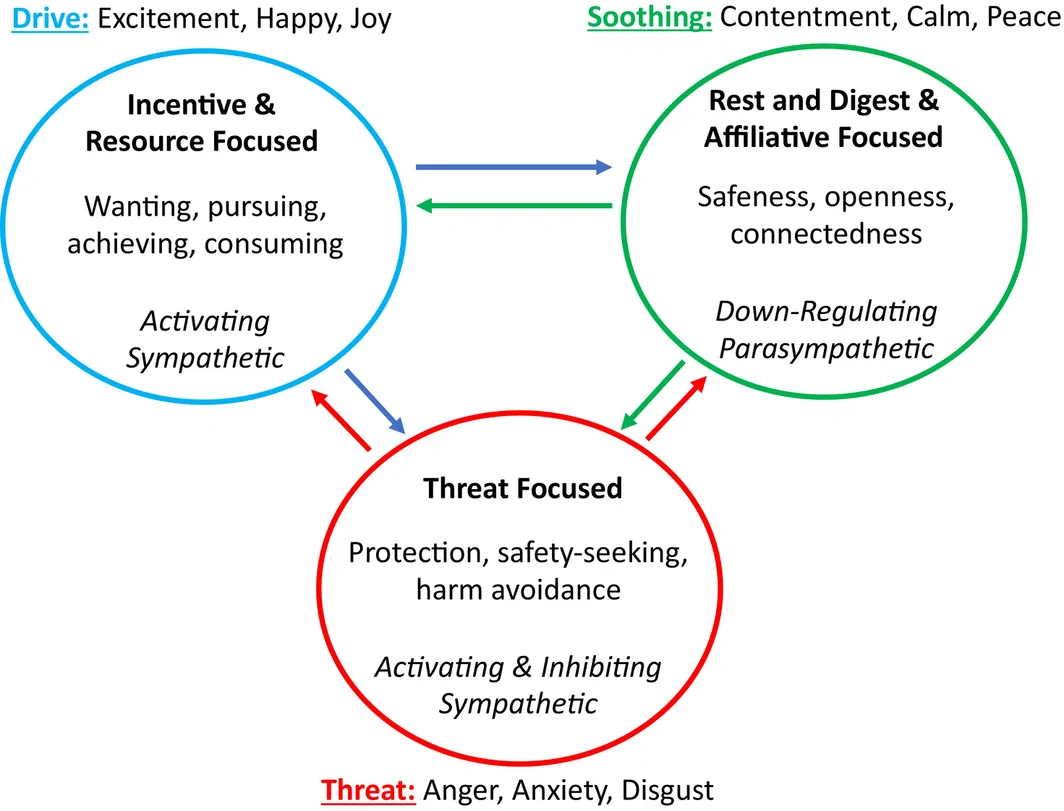
The Tripartite Model of Emotion Regulation: The Three Circles. Note From ‘Essentials of Compassion Focused Therapy: A Practice Manual for Clinicians’ by N. Petrocchi, Kirby, & Baldi, 2024, Oxfordshire, UK: Routledge. Copyright 2024 by Routledge. Adapted from P. Gilbert (2009). Reprinted with permission.

From an evolutionary perspective, human distress often stems from the activation of threat‐focused social rank systems, wherein individuals perceive themselves as subordinate to hostile or dominant others (Gilbert, 2014). In the context of voice hearing, distressing voices frequently simulate these dominant–subordinate dynamics, positioning the hearer in a state of powerlessness, shame and threat‐system hypervigilance (Mayhew & Gilbert, 2008). Traditional efforts to fight or suppress voices often inadvertently reinforce this threat loop. Compassion focused therapy (CFT) offers a novel theoretical framework by targeting the underlying affect‐regulation systems. Rather than attempting to eliminate the voice, CFT helps voice hearers activate their affiliative (soothing) system to establish internal and external safeness (Heriot‐Maitland et al., 2019). By cultivating a compassionate self’, individuals can shift their relational stance towards the voice—transitioning from fearful submission to grounded, compassionate engagement—which reduces the emotional salience, hostility and distress associated with the voice‐hearing experience (Heriot‐Maitland & Levey, 2021; Mayhew & Gilbert, 2008).

Research by Heriot‐Maitland et al. (2019) has been at the forefront of applying this theoretical perspective of CFT to voice‐hearing. The threat system, while being attuned to potential threats in the environment, is also thought to be attuned to dominant and submissive roles within social groups. According to Heriot‐Maitland (2021), fearful psychotic experiences involve dominant–subordinate interactions, which may have been internalised from trauma or previous experience of being harmed by others. Given the high levels of comorbidity of voice hearing to childhood trauma (Peach et al., 2020), where dominant and submissive roles likely played out, this has further predisposed the individual who hears voices to have a further sensitised threat system with difficulties in soothing this system (Heriot‐Maitland et al., 2019). A CFT approach to working with individuals who hear voices thus aims to switch individuals from the automatic action of the threat system and instead be able to voluntarily activate a compassionate understanding and response towards the voices they hear (see Heriot‐Maitland & Levey, 2021, for a case study example).

Empirically, researchers have found that higher levels of compassion have been found to correlate with lower levels of depression, negative beliefs about psychosis and fear of relapse among a variety of psychiatric diagnoses (e.g., Kirby, 2025; Laithwaite et al., 2009; Mayhew & Gilbert, 2008). Importantly, while antipsychotic medication remains a primary treatment component, pharmacological intervention alone is frequently insufficient to achieve full symptom resolution or functional recovery for individuals experiencing psychosis (Harrow & Jobe, 2013). Furthermore, pharmacotherapy is often constrained by sub‐optimal treatment adherence (e.g., García et al., 2016) and significant side‐effect burdens (e.g., Leucht et al., 2013; McEvoy et al., 2007; Waterreus et al., 2012). Consequently, there is a clear need for complementary psychological approaches, such as CFT, which target the affective, relational and shame‐based mechanisms underlying psychotic distress. Based on the emerging empirical studies, there is some evidence for CFT to help those who experience voice hearing in psychosis. For example, a randomised clinical control trial consisting of 40 individuals with a schizophrenia spectrum disorder was allocated to either receiving treatment as usual (TAU) or TAU plus CFT for 16 weeks. Those in the CFT plus TAU were found to have clinically improved symptoms, in addition to decreases in depression, increases in compassion and reductions in perceived social marginalisation, in comparison to those who received TAU alone (Braehler et al., 2013).

### Study aims

A critical aspect of the CFT intervention approach is the psychoeducation of the three circle model, and how CFT aims to develop a greater balance of these systems. As a result, a 5‐min psychoeducation video known as ‘*Compassion for Voices: A Tale of Courage and Hope’* was developed for use as a therapeutic, educational and destigmatising resource by Dr. Charlie Heriot‐Maitland, Kate Anderson and the Cultural Institute at King's College London. The compassion for voices video was designed to be a destigmatising educational and therapeutic tool, yet it has not been explored how the video may be received by voice‐hearers.

Efforts to address the stigma and self‐stigma associated with hearing voices have traditionally relied on public awareness campaigns or biomedical psychoeducation, approaches that have demonstrated limited success in altering internalised shame or lived distress. By contrast, non‐pathologising, community‐led initiatives, most notably Hearing Voices Groups (HVGs), have had significant positive impact in reducing stigma by providing peer‐supported spaces that normalise voice hearing and honour the personal meaning of these experiences (Corstens et al., 2014; Longden et al., 2018). Building upon this shift towards acceptance and relational reframing, the aim of the project is to survey people who hear voices to understand their impressions of the compassion for voices video. The survey will explore the following questions: (1) what are the reactions/responses of people who hear voices to the video; and (2) to what extent do people who hear voices think that the video would impact areas in health service delivery and society? As a result, our survey is explorative and qualitative, as we aim to understand whether the psychoeducational video resource, ‘Compassion for Voices: A Tale of Courage and Hope’ can destigmatise attitudes towards voice hearing.

## METHOD

### Participants

The sample was drawn from a larger online study (N=141) examining responses to the Compassion for Voices animated film across various stakeholder groups (e.g., mental health professionals, family members, general public). Participants were recruited via convenience sampling using an anonymous online survey link distributed alongside the film across online platforms, community groups, and the researchers' academic and professional networks. To maintain complete participant anonymity, minimise survey length and align with a non‐pathologising approach centred on self‐reported lived experience, formal demographic data (e.g., sex, age, clinical diagnoses, treatment history) were not collected. The only demographic variables collected were whether participants identified as a person who hears voices, a family member/friend of a voice hearer, a mental health professional working with voice hearers, a clinician in general mental health services, a policy decision‐maker or a member of the general public. For the current study, analysis was restricted to the subsample of participants who self‐identified as having lived experience of hearing voices (*n* = 20). Ethical approval was granted by The University of Queensland Institutional Human Research Ethics Committee (HE000262) to retrospectively examine the annonymously collected data.

### Procedure

Participants viewed the animated video, ‘Compassion for Voices: A Tale of Courage and Hope’ (5 min 13 s). The film depicts the story of ‘Stuart’, an individual experiencing distressing auditory verbal hallucinations (voices). Framed through the theoretical lens of CFT and the Three Circles model of affect regulation, the animation illustrates how threat‐system overactivation and intense self‐criticism exacerbate distress and relational subordination to hostile voices. The narrative follows Stuart's engagement in compassionate mind training, illustrating key CFT practices including body‐based grounding, soothing rhythm breathing, compassionate imagery and embodied practice of the ‘compassionate self’. Through the activation of his soothing/affiliative system, Stuart learns to establish internal safeness and initiate a compassionate dialogue with his critical voices. Rather than aiming for symptom elimination or voice suppression, the film normalises voice hearing as a meaningful human experience linked to past adversity and illustrates a relational shift from threat‐based fear and submission to compassionate acceptance, agency and social reconnection. Below in Figure 2, two screengrabs are provided showing the experience of Stuart and his three affect‐regulation systems, and the video can be accessed here: https://www.youtube.com/watch?v=VRqI4lxuXAw.

**FIGURE 2:**
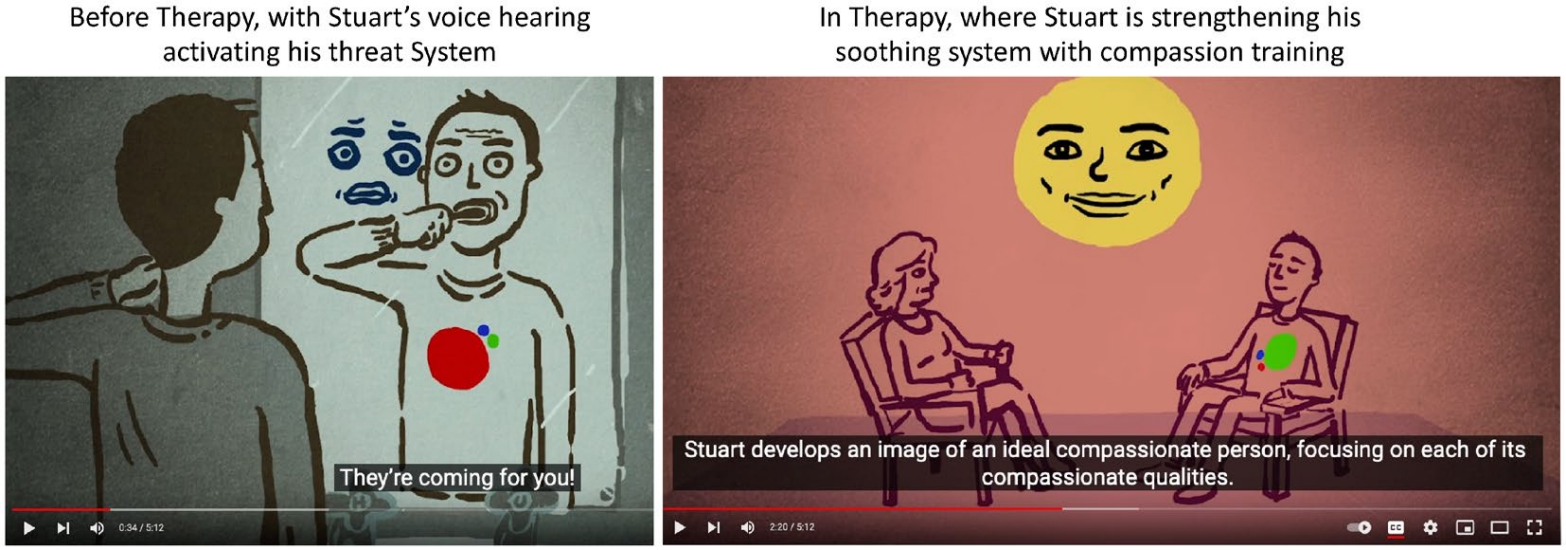
Clips from the Stuart video showing his voice hearing and therapy, and how they activate his three affect‐regulation systems.

The video was developed by Dr. Charlie Heriot‐Maitland and colleagues (Cultural Institute at King's, 2015), who used co‐production principles to increase the sense of engagement and connection of the video for those who experience hearing voices. Thus, those with a lived experience of voice hearing were included as part of the development of the material in the video. In addition, Dr. Eleanor Longden, who has lived experience of voice hearing and who is an advocate for psychosis was the narrator, and ‘Stuart’ was voiced by Rufus May, an advocate for voice hearing and psychosis who also has a lived experience.

### Measures

A bespoke self‐report survey was designed specifically for the purpose of this project. The only demographic questions asked were whether participants self‐identified as a person who heard voices, were a friend or family member of someone who heard voices, worked with people who heard voices, worked in other areas of mental health services, were someone who could influence policy or were a member of the general public. As mentioned, our study is solely focused on those who hear voices. An open response question was used for participants to describe the main impact of the film. The questionnaire also contained yes/no questions asking if participants believed the film could impact a variety of areas, followed by open response questions to allow for further elaboration on the response. A final question was used for participants to provide any other feedback. All questions used in the bespoke survey can be seen in Table 1.

**TABLE 1:** Bespoke Survey Questions and Response Types.

Question	Response type
Please describe what, for you, has been the main impact of this film? (e.g. has it changed your understanding about voices? has it changed your attitude or approach towards people who hear voices? has it encouraged you to think about, or to do, something in a different way?). Do you think this film has, or could potentially have, an impact in the following areas?	Open ended
Impact on health and welfare	Yes/No; Open ended
b Impact on health service policy	Yes/No; Open ended
c Impact on health service provision	Yes/No; Open ended
d Impact on non‐health service provision	Yes/No; Open ended
e Impact on public understanding	Yes/No; Open ended
f Impact on economic	Yes/No; Open ended
g Impact on social/society	Yes/No; Open ended
3 Please use this space to add any additional feedback/comments/thoughts	Open ended

### Design and data‐analytical approach

The design of the study was exploratory and qualitative in nature; thus, we used thematic analysis outlined by Braun and Clarke (2006). The analysis began with repeated reading of the participant responses and the generation of initial codes. The analysis was then conducted across all open‐ended questions as detailed in Table 1 (rather than each question individually) to manage the overlap across questions. Initial codes were collated, developed and refined into themes that represented consistent patterns across the data set. Themes were generated on the basis of how often they occurred across participants and through analytical decisions based on whether a theme ‘captures something important in relation to the overall research question’ (Braun & Clarke, 2006, p. 82). The relevance of each theme was checked against the coded extracts and organised under superordinate headings. A written narrative report was then developed, summarising the findings with exemplar quotations from the raw data.

Thematic analysis of the qualitative data was independently completed by the second author, with supervision from the first author. All data extracts supporting each theme were recorded and provided to collaborator (the second author) to review to ensure that the themes were justified and grounded in the qualitative data.

In terms of reflexivity, all authors practice CFT and have been trained by Professor Paul Gilbert (founder of the modality). We acknowledge a shared assumption that a destigmatised view when learning of the three affect‐regulation model is an expected outcome of the psychoeducation material in CFT.

## RESULTS

### Thematic analysis

Among the 20 participants, the total number of words used in answer to the open questions ranged from 1 to 129 words, with an average response of 20.34 words. The analysis produced three inter‐related superordinate themes which related to each research question, as can be seen in Table 2.

**TABLE 2:** Superordinate Themes and Subthemes (number of participants for whom this meaning‐unit occurred).

Superordinate theme	Subtheme
A sense of shared identity to Stuart's journey	Hearing voices is a shared universal experience (6) Accurate representation of lived experience (11)
2 CFT approach desired, but recognition of fears, blocks and resistances to developing compassion	CFT is an acceptable intervention (6) CFT to be integrated with existing interventions (7) Difficulties building compassion (3)
3 Stuart's psychoeducation video as validating, destigmatising and hopeful	Reducing stigma and normalising experience (14) Increasing compassion from others (12) Giving hope (10)

### A sense of shared identity to Stuart's journey

Two themes emerged in relation to participants’ views on the compassion for voices video. The first theme identified was voices being a shared or universal experience, demonstrated through responses such as ‘*Everyone hears voices. I guess the difference between being psychotic and not is the severity. I know my experience. Each one of us hears voices’*, *‘the film was very good for me to recognise that other people have these thoughts too and can manage them’* and ‘*we all have voices. The quality of voices vary from a person*[‘s] *experience’*. The second theme that emerged in response to the question of participants' views of the compassion for voices video was that it is an accurate representation of their lived experience, as seen through responses such as *‘the voices were eerily similar to the types I hear. Thank you for the film. It has really helped’*, *‘this video shows exactly what my voices are’* and *‘Oh my God. I can't believe the video. It is EXACTLY what goes on in my head. This is the FIRST time I have EVER come across something that nails my experience’*. However, one participant shared a dissenting opinion: *‘the video does not distinguish between internal dialogue voices and the literally, audible outside voices, or the voice like mine, an intruder with a presence, messages and a personality outside of my usual self but without audible words’*.

### CFT approach desired, but recognition of fears, blocks and resistances to developing compassion

Three themes were connected to the superordinate theme of desiring the integration of CFT into current therapy, however, recognising the difficulties in developing compassion. The first theme that emerged was CFT being seen as an acceptable intervention, with participants providing statements such as *‘I would like to learn more to be compassionate and understand my critical voices more so they won't have such an impact on my life and I can move forward more’* and *‘made me want to work on creating a compassionate voice’*. A second theme emerged with participants viewing CFT as a possible alternative or adjunct to the use of medication, with statements such as ‘*alternatives to strict pharmacological approach’* and ‘*including psychology along with psychiatry*’. One participant had a dissenting view, expressing views that they did not believe psychological interventions would be added to treatment. A third theme emerged from participants' responses relating to the difficulties with developing compassion, with such responses as *‘It encapsulates how I feel about my personal experience. But it conveys little of the panic that can occur when compassion appears impossible’* and *‘as an art student and person who hears voices and struggles with them this film has made me understand in a positive way how to be compassionate to my voices but it's for me a struggle and still have not overcome but the film in an easier understanding way to be nice to myself and my voices’*.

### Stuart's psychoeducation video as validating, destigmatising and hopeful

Three themes emerged for this superordinate theme. The first theme that emerged was reducing stigma and normalising the experience of hearing voices. A number of participants provided statements directly endorsing normalising their experience (e.g., ‘*this helps normalize the experience’*) and reducing social stigma (‘*less social stigma and misunderstandings*’), with others expanded further, suggesting that the hearing voices video may be useful for providing psychoeducation and promoting understanding in the broader population ‘*for the general public who fear people who hear voices this is a good educational film that would help the general population have a more compassionate understanding to people who hear voices*’. Other participants commented on the spectrum of experiences of hearing voices (e.g., ‘*more acceptance of voice hearing as a phenomenon*’) and noted that reduction in stigma may increase help‐seeking behaviour (‘*reduce the stigma and make people able to ask for help*’) and increase community participation (‘*if managed well work can continue*’; ‘*that people would not be in isolation secluded but become a[n] active part of the community they live*’).

The second theme that emerged regarding the third research question was increasing compassion from others. Participants responded indicating they thought the video could increase compassion from service providers (‘*It can encourage people to take compassion to heart when offering services*’) and policy makers (‘*It can help policymakers see things from another perspective’*). Additionally, participants thought the video may be helpful for increasing compassion from the general public towards those who hear voices (*‘Can help people appreciate the challenges voice‐hearers experience, that treating them with compassion makes a difference, and it also normalizes the hearing voices experience’*).

The third theme that emerged regarding the third research question was providing hope. Participants viewed the compassion for voices video as containing a message of hope (e.g., ‘*Give first hope’; ‘there is a message of hope in this video as well, and I'm eager to learn more about how to guide others along the way through their recovery through compassion’*.), including providing hope that they can live a meaningful life with their voices (e.g., ‘*Help me live a life I'd want to be alive in*’: *‘had a psychiatrist who told me to live with it. This will help me live with it*’). Participants also identified that the compassion for voices video helped them to feel less alone (e.g., ‘*the film was very good for me to recognise that other people have these thoughts to and can manage them’; it has encouraged me to continue practicing on accepting the voices. It has also made me understand that I'm not the only one and that I can handle it*). Participants also identified acceptance of themselves and their voices from the compassion for voices video (e.g., ‘*acceptance of self‐ less anxiety, depression etc’; ‘to recognize and accept the voices reduces internal resistance resulting to a healthier living’; ‘usually the voices are mean and threatening and I wonder why I deserve so much pain. This film has shown me that I'm basically a good person*’).

### Broader health impact of the Stuart video: Descriptive statistics

We also examined, descriptively, the perceived impact the compassion for voices video could have on a broad range of social and health areas. Table 3 outlines this descriptive data, with public understanding and broader society emerging as the areas in which participants believed the compassion for voices video could have the greatest impact, with 18 participants (90%) indicating yes to positive impact. The majority of participants (85%) also indicated that the compassion for voices video could positively impact on people's health and wellbeing. Conversely, the majority of participants felt the compassion for voices video was least likely to have an impact economically.

**TABLE 3:** Participants responses to areas of impact.

Area of impact	Yes	No	Left blank
Health and welfare	17 (85%)	2 (10%)	1 (5%)
Health service policy	13 (65%)	4 (20%)	3 (15%)
Health service provision	12 (60%)	4 (20%)	4 (20%)
Other (non‐health) service provision	9 (45%)	5 (25%)	6 (30%)
Public understanding	18 (90%)	1 (5%)	1 (5%)
Economic impact	6 (30%)	9 (45%)	5 (25%)
Society/Social	18 (90%)	1 (5%)	1 (5%)

## DISCUSSION

The aim of the present study was to explore the views of individuals who hear voices on the compassion for voices—‘Stuart’—video, which was specifically designed to be destigmatising and as psychoeducational tool on CFT. Overall, there were three superordinate themes. First, ‘*A Sense of Shared Identity to Stuart's Journey’*, participants identified hearing voices to be a universal experience, which is consistent with previously mentioned research identifying that voice hearing is a relatively common experience across cultures and occurs in non‐clinical populations (e.g., Beaven et al., 2011). Participants also identified the Stuart video to be an accurate representation of their experiences. However, of note was one participant who provided a dissenting view of the Stuart video accurately describing their experiences. Although the participants for this study all indicated that they had a lived experience of hearing voices, it is possible that the hearing of voices was not their primary symptom of psychosis. As previously discussed, voice hearing is a varied phenomenon that occurs in non‐clinical populations, as well as across diagnostic categories. It is worth noting that the current study did not differentiate phenomenology of voice hearing nor content heard. Thus, the Stuart video may not accurately represent all voice hearing experiences (for example, command auditory hallucinations were not portrayed). Importantly though, given the majority of participants with a lived experience of hearing voices connected with the video, it indicates that this video is a useful resource for clinicians when applying CFT to those who hear voices. In addition, it also highlights the importance of co‐production principles (see Schouten et al., 2022), as those with a lived experience of voice hearing helped in the development of the video, as well as providing the narration, which may have led to participants feeling a sense of shared connection.

The second superordinate theme was *‘CFT approach desired, but recognition of fears, blocks and resistances to developing compassion’*. Consistent with past literature, CFT was viewed as an acceptable intervention (e.g., McManus et al., 2018); however, participants also noted difficulties with developing self‐compassion. This is consistent with previous research on CFT, with fears, blocks and resistances to developing self‐compassion well documented and even expected (e.g., Gilbert & Mascaro, 2017). Participants expressed a desire for integration of CFT into management of their illness, but also expressed they felt it was unlikely to be adopted by services due to funding constraints and predominance of a medical model of treatment. For example, while cognitive behavioural therapy for psychosis (CBT‐p) has largely reported positive outcomes in symptom severity and improvement in psychological health, has low dropout rates and is generally found to be acceptable by participants (Bird et al., 2010; Peters et al., 2015; Wood et al., 2013), barriers to inclusion in services have been identified, including high costs related to staff development and training (Roth & Pilling, 2013) and lack of prioritisation in funding (MIND, 2010), with generally low uptake by existing services (Colling et al., 2017). However, a more recent evaluation of improving access to therapy for those living with severe mental illness found implementation into existing psychosis services may be feasible (Johns et al., 2019).

The third superordinate theme was ‘*Stuart's psychoeducational video as validating, destigmatising and hopeful*’. Participants viewed the video as having the potential to reduce the stigma they experienced and normalise their experiences in broader society, leading to increased compassion from others. Participants were hopeful that this may be helpful in increasing engagement in meaningful activities, including employment and community engagement, as well as maintaining housing. Participants also saw the Stuart video as potentially being helpful in shifting policy and service development. Additionally, participants identified a message of hope from Stuart in such that there were reductions in negative beliefs about themselves and increasing belief that a meaningful life could be built while living with voices. Altogether, participants responses in this superordinate theme pointed towards a desire for understanding and compassion from others, with hope for developing a more meaningful life. This is consistent with a recovery‐oriented model of service delivery, which promotes building a meaningful life beyond living with a mental illness (Andresen et al., 2003). While a biomedical model tends to prevail in service delivery (Morera et al., 2017), including functional recovery such as return to work, study or increased social connection is linked to better long‐term outcomes such as higher rates of remission and better psychosocial functioning (Álvarez‐Jiménez et al., 2012; Frawley et al., 2023).

Importantly, the results from this study indicate that the primary impact reported by voice hearers in this brief intervention was validation, normalisation and identifying the film as a powerful tool for public education, rather than a standalone therapeutic solution for voice‐hearing distress. We view this as an important nuance: A 5‐min animated video serves primarily as a psychoeducational and destigmatising ‘doorway’ that fosters social safeness and hope, rather than replacing comprehensive therapeutic intervention, such as full CFT intervention. While the data of the current study support the use of Stuart video as being helpful for psychoeducation and as a tool for destigmatising hearing voices, there are some potential limitations to the present study worth noting. Firstly, only minimal demographic information was collected on participants and our sample only consisted of 20 participants. For instance, there were no data collected on cultural background, length of hearing voices or previous access to treatment. Thus, there may be questions as to the generalisability of our sample and consideration may be given to a repeat of the current study with both a larger sample and more demographic information to ensure generalisability to a broader population, as one participant dissented to the video being an accurate representation of their lived experience of voice hearing. Secondly, the current study only represents an initial study on use of the Stuart video as a possible destigmatising tool and to provide education on CFT as an intervention for those who hear voices. Further research is needed to explore the viability of using CFT in psychosis services and to support CFT as an evidence‐based treatment for hearing voices, and whether viewing the Stuart video would have an impact upon negative perceptions of hearing voices among the general population. Although we examined whether the Stuart video could have a positive impact on a range of broad social and health areas, we did not examine this statistically. Moreover, we did not collect data on what the positive impact would look like, which could be helpful in designing the next series of studies aimed to examine the impact of the video. Critically, this study was focused on those currently hearing voices, but it would be important to know how other health professionals and family members viewed this resource, as these factors have been shown to be important for engagement with health services and outcomes (e.g., Luckstead et al., 2018).

### Clinical implications

Using the Stuart video can be helpful for clinicians to use in the therapy session itself to describe the conceptual model of CFT, as well as answer any immediate questions the client might have. This might be a more interesting and engaging way to provide psychoeducation than to be told didactically from the clinician. In addition, clients can re‐watch the video in their own time, allowing for the themes of the material to be better absorbed rather than only relying on hearing it once from the therapist. Thus, it can be referred to easily within the sessions to ask questions and explore themes of the three affect‐regulation systems model and CFT.

The theme of hope is critical. It is not uncommon with severe mental illness for the client to lose hope (Barut et al., 2016) and close family members or friends to experience caregiver fatigue and burnout, including feelings of hopelessness (Brain et al., 2018). Though hope is associated with increased quality of life and considered an important factor for recovery, it is understandably difficult to access for both the individual and families living with severe mental illness (Hayes et al., 2017). In these instances, it is suggested the therapist may need to be the provider of hope (Sari et al., 2021). Thus, for participants who are voice‐hearers to see a sense of hope from this short 5‐min video is potentially very powerful. Clinicians can potentially use this video to help facilitate hope and show that a meaningful life while living with mental illness can be built.

## CONCLUSION

This study was conducted as part of a broader research project, focussing only on people who identified with hearing voices. It is also the first project to explore the use of and the views of those who hear voices on the Stuart video. Overall, there is preliminary evidence for the use of the video as both a tool for destigmatising hearing voices and for acceptability of CFT in treating individuals who hear voices; however, further research is needed to explore and confirm findings.

## AUTHOR CONTRIBUTIONS

**Kaedee Abel:** Conceptualization; writing – original draft; methodology; writing – review and editing; visualization; formal analysis. **Charles Heriot‐Maitland:** Conceptualization; writing – review and editing; investigation. **James N. Kirby:** Conceptualization; writing – review and editing; writing – original draft.

## FUNDING INFORMATION

Video production was funded by Compassionate Mind Foundation, Balanced Minds, Medical Research Council and King's College London.

## CONFLICT OF INTEREST STATEMENT

James N. Kirby is a co‐author of ‘Essentials of Compassion Focused Therapy: A Practice Manual for Clinicians’.

## Data Availability

The data that support the findings of this study are available on request from the corresponding author. The data are not publicly available due to privacy or ethical restrictions.
